# Supplementary material for: Dual roles of neutrophils in metastatic colonization are governed by the host NK cell status
Source: Nat Commun. 2020 Sep 1;11:4387. doi: 10.1038/s41467-020-18125-0 (PMC7463263; doi:10.1038/s41467-020-18125-0)
Supplement: Supplementary file 1 — Supplementary Information [file 41467_2020_18125_MOESM1_ESM.pdf]

Supplementary Information

Dual roles of neutrophils in metastatic colonization are governed by the host NK cell status

Li, Lu et al.

Supplementary materials include:

Supplementary Figures 1 to 11

Supplementary Table 1

Supplementary References

Supplementary Figures

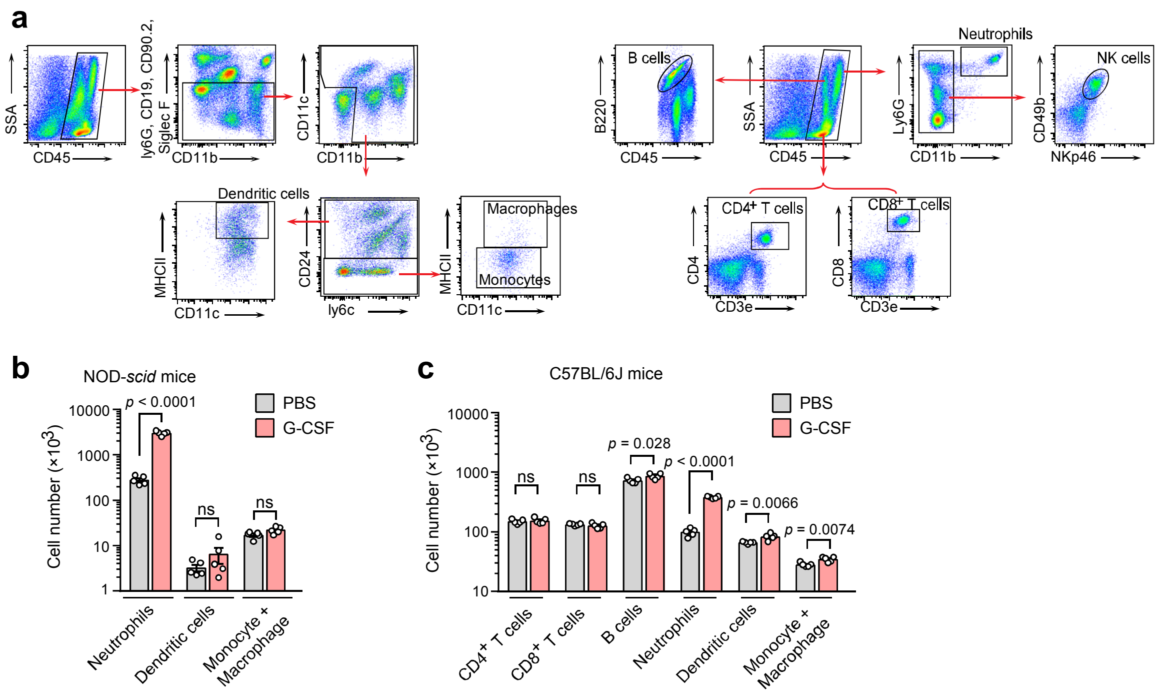

d

Strain name	NOD-*scid*	NSG
Neutrophil	Present	Present
Natural killer (NK) cells	Present	Absent
Mature B cells	Absent	Absent
Mature T cells	Absent	Absent
Dendritic cells	Defective	Defective
Macrophages	Defective	Defective

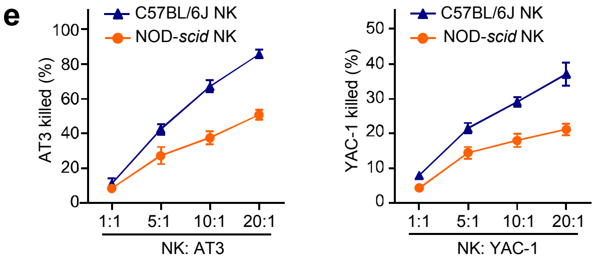

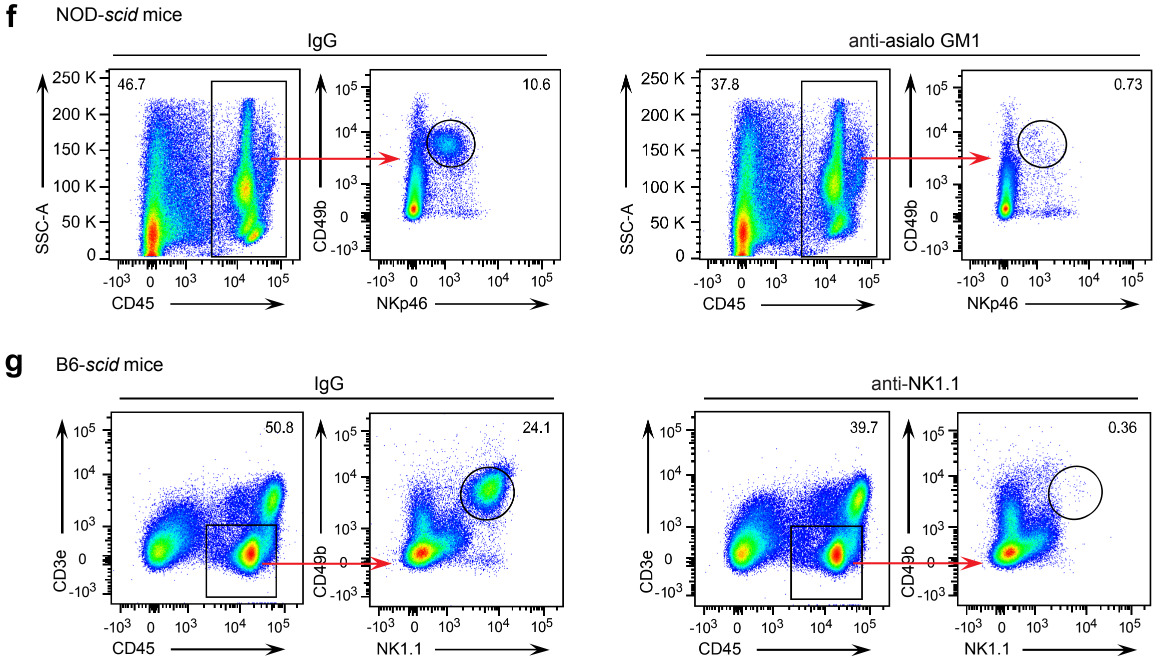

Supplementary Figure 1. Exogenous G-CSF injection caused a specific neutrophil expansion in the recipient mouse lungs

a-**c**, NOD-*scid* mice (**b**) or C57BL/6J mice (**c**) received intraperitoneal (i.p.) injection of recombinant mouse G-CSF (2.5 μg per mouse). 3 days later, the mouse lungs were isolated for total cell counts of the indicated immune cells as determined by flow cytometry. The flow cytometry gating strategy was shown in (**a**). Data are represented as mean ± SEM. n = 5 mice per group. *P* values were determined by unpaired two-tailed *t*-test. ns, not significant.

d, A comparison of the immune system between NOD-*scid* and NSG mice, based on previous publications[1, 2].

e, NK cells isolated from spleens of naïve C57BL/6J or NOD-*scid* mice were subjected to an *ex vivo* cytotoxicity assay with AT3 cells (left) or YAC-1 cells (right) as target cells. Data are represented as mean ± SD of 4 biologically independent cell cultures.

f-**g**, NK cells were depleted in NOD-*scid* mice (**f**) and B6-*scid* mice (**g**) by i.p. injection of anti-Asialo GM1 (25 μl per mouse) and anti-NK1.1 (25 μg per mouse) respectively, every 3 days for 3 times. The depletion efficiencies were then determined by measuring the $CD45^+CD49b^+NKp46^+$ and $CD45^+CD49b^+NK1.1^+$ cells in NOD-*scid* and B6-*scid* mice respectively by flow cytometry. Representative flow cytometry profiles (n = 4 mice per group) from three independent experiments are shown.

Source data are provided as a Source Data file.

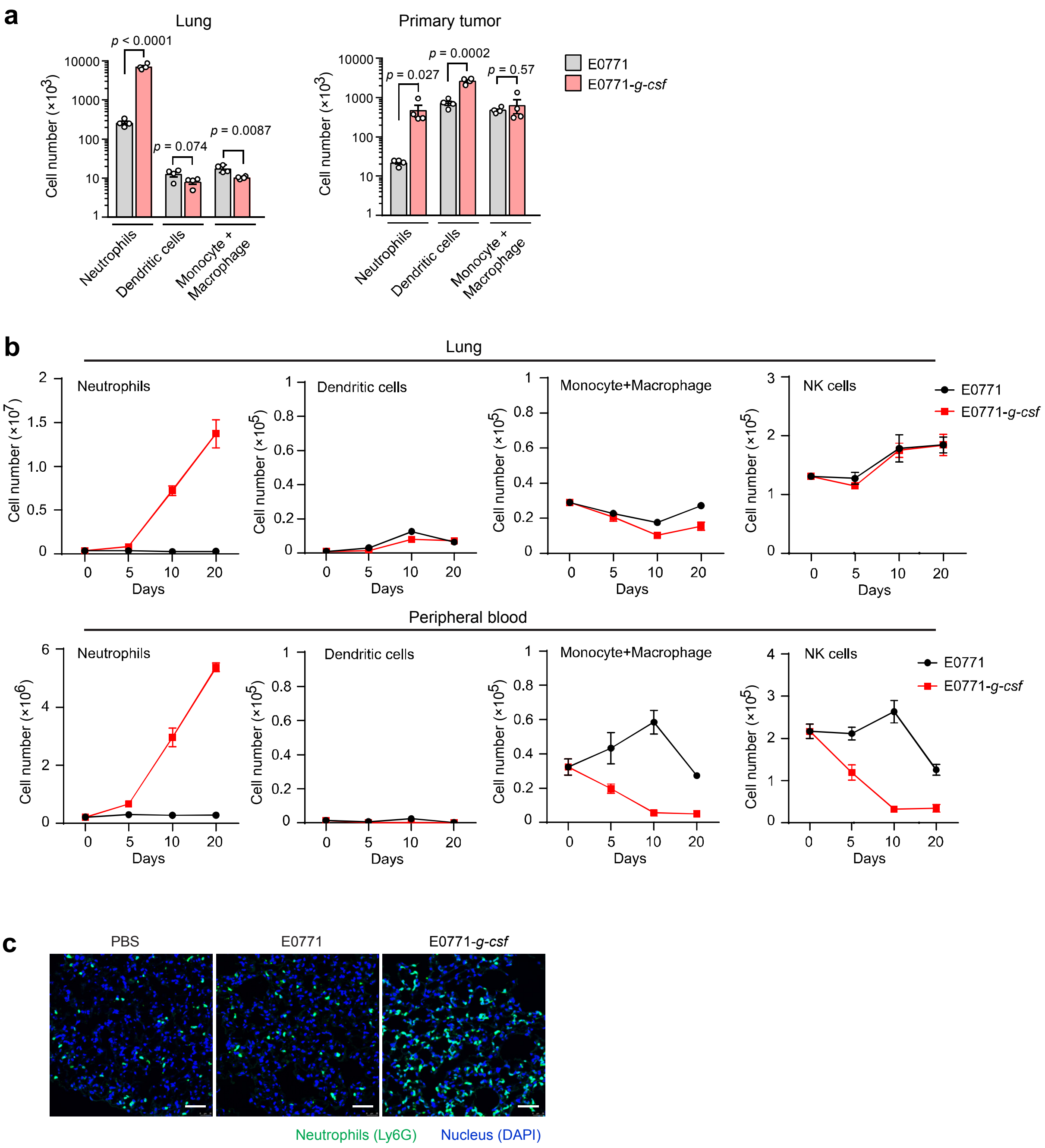

Supplementary Figure 2. The impact of tumor cell *G-csf* overexpression on the host immune cell subsets

NOD-*scid* mice were orthotopically injected with E0771 or E0771-*g-csf* cells to generate the non-inflammatory or inflammatory tumor-bearing host conditions, respectively. (**a**) At the pre-metastatic stage (day 10), the frequencies of indicated immune cell subsets in mouse lungs and primary tumors were determined by flow cytometry and the total cell counts were then

calculated. (**b**) To delineate the dynamic of immune profiles in the E0771/E0771-*g-csf* models, the mouse lungs and peripheral blood (0.5 ml) were collected on day 0 (naïve mice control), 5, 10 and 20. The frequencies of indicated immune cell subsets were determined by flow cytometry and the total cell counts were then calculated. The data on day 0 were acquired from the same group of naïve mice. (**c**) To determine the neutrophil infiltration in the pre-metastatic lungs in situ, the mouse lungs were freshly isolated on day 10 and sectioned for immunostaining of neutrophils (Ly6G). Representative pictures from 4 mice per group are shown. Scale bars, 50 μm.

Data are represented as mean ± SEM (n = 4 mice per group) for (**a**) and (**b**). *P* values were determined by unpaired two-tailed *t*-test. Source data are provided as a Source Data file.

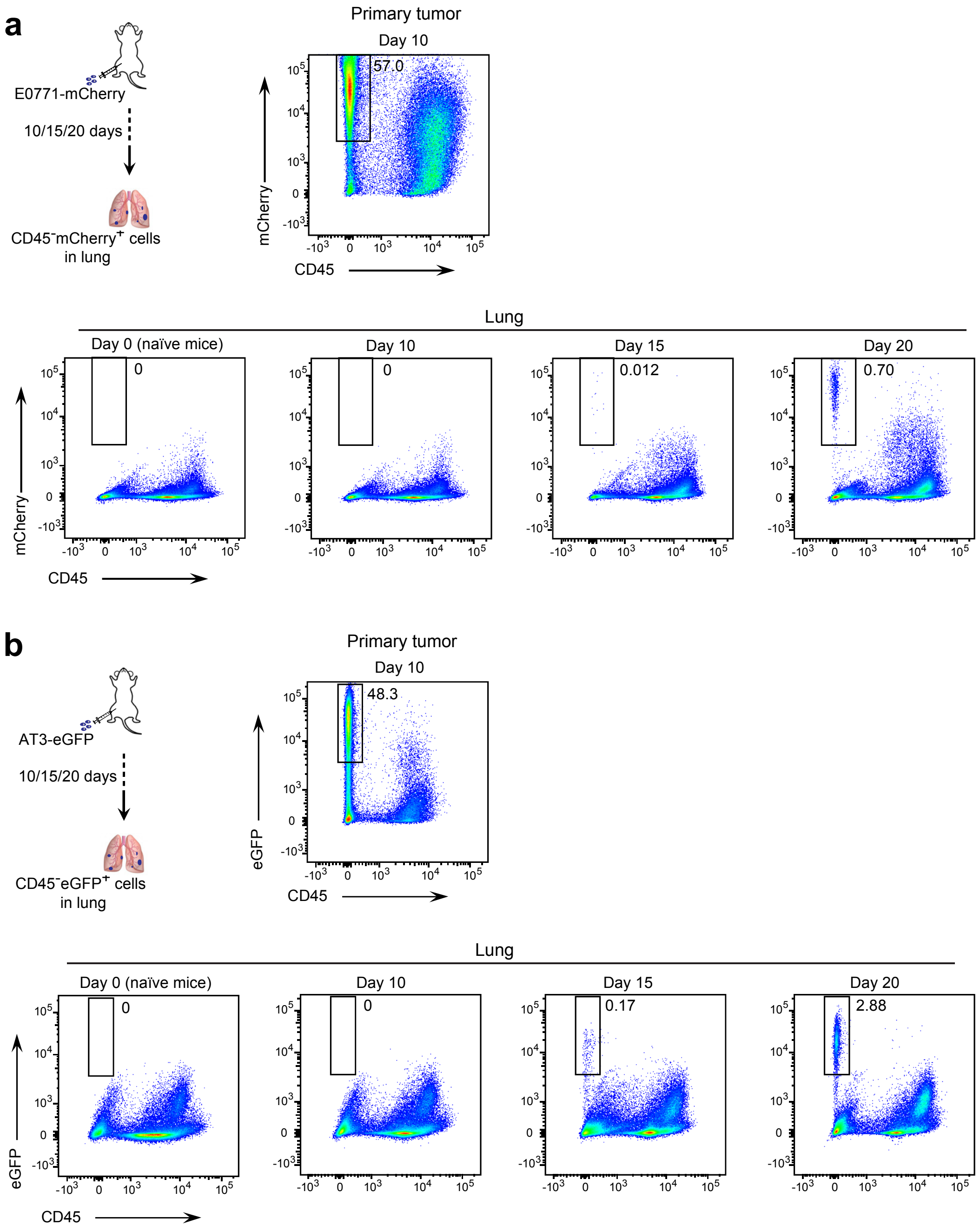

Supplementary Figure 3. Determination of the pre-metastatic time windows in the E0771 and AT3 models

NSG mice were orthotopically implanted with E0771-mCherry (**a**) or AT3-eGFP cells (**b**). On days 10, 15 and 20, the percentages of tumor cells in the mouse lungs were assessed by flow cytometry and representative plots from 5 mice per group are shown. Primary tumor samples and naïve mice-derived lung samples were set as positive and negative controls, respectively. Two independent experiments were performed and similar results were obtained.

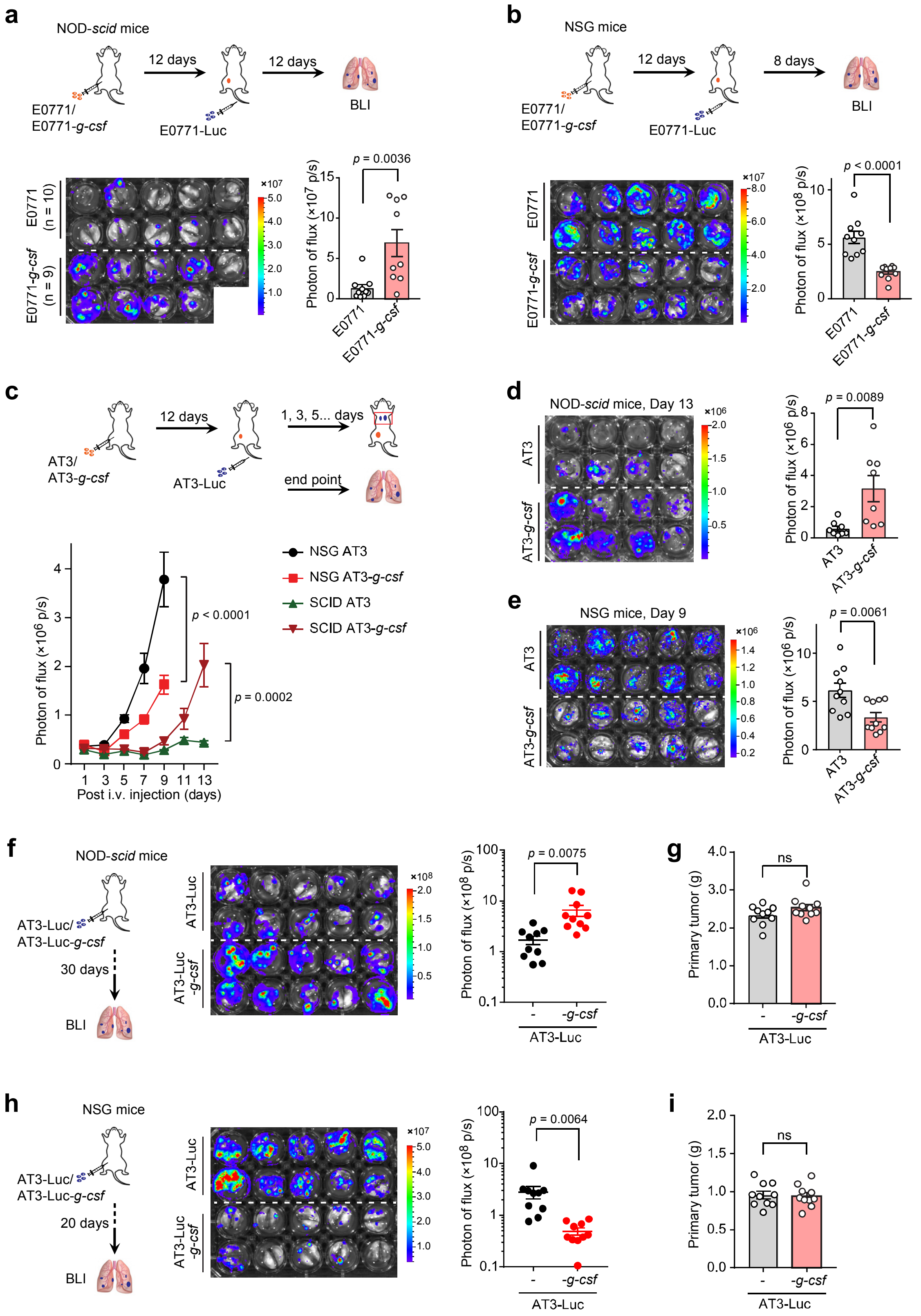
a
NOD-scid mice
12 days
12 days
E0771/
E0771-g-csf
E0771-Luc
BLI
E0771 (n = 10)
E0771-g-csf (n = 9)
p = 0.0036
Photon of flux (×10^7 p/s)
E0771
E0771-g-csf
b
NSG mice
12 days
8 days
E0771/
E0771-g-csf
E0771-Luc
BLI
p < 0.0001
Photon of flux (×10^8 p/s)
c
12 days
1, 3, 5... days
AT3/
AT3-g-csf
AT3-Luc
end point
NSG AT3
NSG AT3-g-csf
SCID AT3
SCID AT3-g-csf
p < 0.0001
p = 0.0002
Photon of flux (×10^6 p/s)
Post i.v. injection (days)
d
NOD-scid mice, Day 13
AT3
AT3-g-csf
p = 0.0089
e
NSG mice, Day 9
p = 0.0061
f
NOD-scid mice
AT3-Luc/
AT3-Luc-g-csf
30 days
BLI
AT3-Luc
AT3-Luc -g-csf
p = 0.0075
Photon of flux (×10^8 p/s)
g
ns
Primary tumor (g)
h
NSG mice
20 days
p = 0.0064
i
ns

Supplementary Figure 4. Tumor cell *G-csf* overexpression led to their increased metastatic colonization in NOD-*scid* mice, but decreased metastatic colonization in NSG mice

a-b, As depicted in top panels, NOD-*scid* mice (**a**) and NSG mice (**b**) were first orthotopically injected with unlabeled E0771 or E0771-*g-csf* cells to generate the non-inflammatory or neutrophil^{high} inflammatory host conditions, respectively. Luciferase-labeled E0771-Luc cells were then i.v. infused on day 12, a time point within the pre-metastatic stage for the orthotopic tumors. At the endpoint, the metastatic progression of E0771-Luc cells in the lungs was detected by *ex vivo* BLI. The endpoint bioluminescence images and the quantification of photon flux of lungs are shown. For NOD-*scid* mice (**a**), n = 10 (E0771 group) and n = 9 (E0771-*g-csf* group). For NSG mice (**b**), n = 10 in each group.

c-e, As depicted in (**c**), NOD-*scid* and NSG mice were first orthotopically injected with unlabeled AT3 or AT3-*g-csf* cells to generate the non-inflammatory or neutrophil^{high} inflammatory host conditions, respectively. Luciferase-labeled AT3-Luc cells were then i.v. infused at the pre-metastatic stage (day 12). The metastatic progression of AT3-Luc cells was then monitored by BLI (**c**). The endpoint bioluminescence images and the quantification of photon flux of lungs are also shown (**d** and **e**). n = 10 mice per group in (**c**), n = 8 mice per group in (**d**) and n = 10 mice per group in (**e**).

f-**i**, As depicted in left panels, NOD-*scid* mice (**f-g**) and NSG mice (**h-i**) were orthotopically implanted with AT3-Luc or AT3-Luc-*g-csf* cells. At the endpoint (day 30 for NOD-*scid* and day 20 for NSG mice), the spontaneous lung metastases were assessed by *ex vivo* BLI (**f** and **h**), and the primary tumors were also dissected and weighted (**g** and **i**). n = 10 mice per group. Data are represented as mean ± SEM. *P* values were determined by unpaired two-tailed *t*-test except (**c**) by two-way ANOVA. ns, not significant. Source data are provided as a Source Data file.

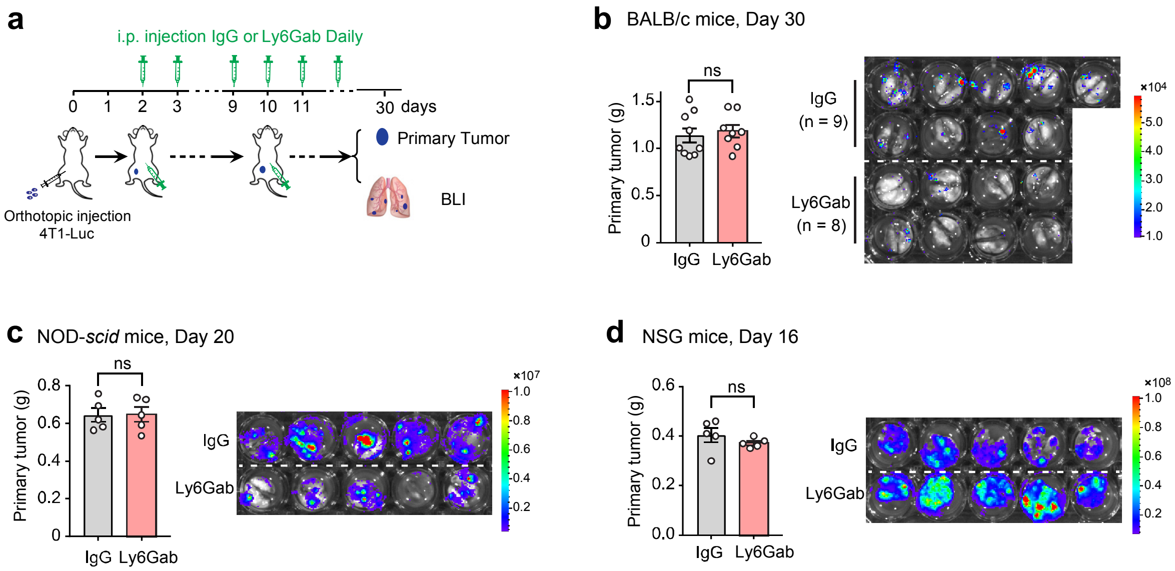

Supplementary Figure 5. Neutrophil depletion showed anti-metastatic and pro-metastatic effects in NK-cell competent and NK cell-deficient mice, respectively, in the 4T1 spontaneous metastasis model

As depicted in (**a**), BALB/c (**b**), NOD-*scid* (**c**) and NSG mice (**d**) were first orthotopically implanted with 4T1-Luc cells, and neutrophils were then depleted in these mice by i.p. injection of anti-Ly6G (12.5 μg per mouse) daily starting from day 2. At the endpoint, the primary tumors were dissected and weighed (left), and the spontaneous lung metastases were examined by *ex vivo* BLI (right). Quantification of photon flux of lungs was shown in Figure 4j. n = 9 (IgG group) and 8 (Ly6Gab group) for BALB/c mice (**b**); and n = 5 per group for NOD-*scid* (**c**) and NSG mice (**d**).

Data are represented as mean ± SEM. Statistical significance was determined by unpaired two-tailed *t*-test. ns, not significant. Source data are provided as a Source Data file.

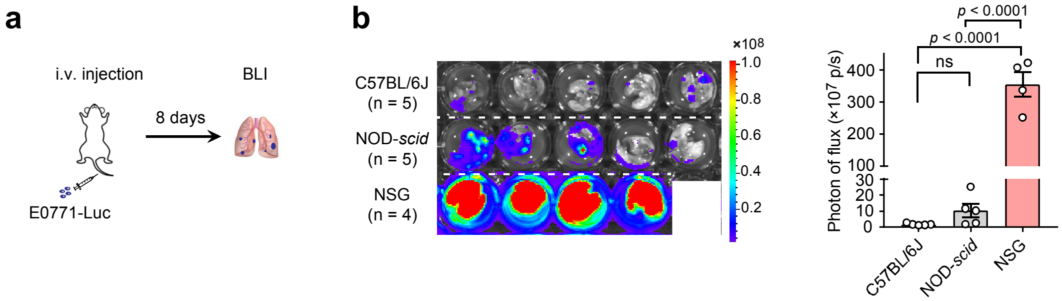

Supplementary Figure 6. NK cells play a primary role in restricting early lung metastasis

As depicted in (**a**), E0771-Luc cells were i.v. injected in C57BL/6J, NOD-*scid* and NSG mice. Eight days later, the lung metastases in each strain of mice were measured by *ex vivo* BLI. The BLI images (**b**, left) and the quantification of photon flux of lungs (**b**, right) are also shown. Data are represented as mean ± SEM (n = 5 for C57BL/6J mice and NOD-*scid* mice, n = 4 for NSG mice). *P* values were determined by one-way ANOVA with Tukey's multiple comparisons test. ns, not significant. Two independent experiments were performed and similar results were obtained. Source data are provided as a Source Data file.

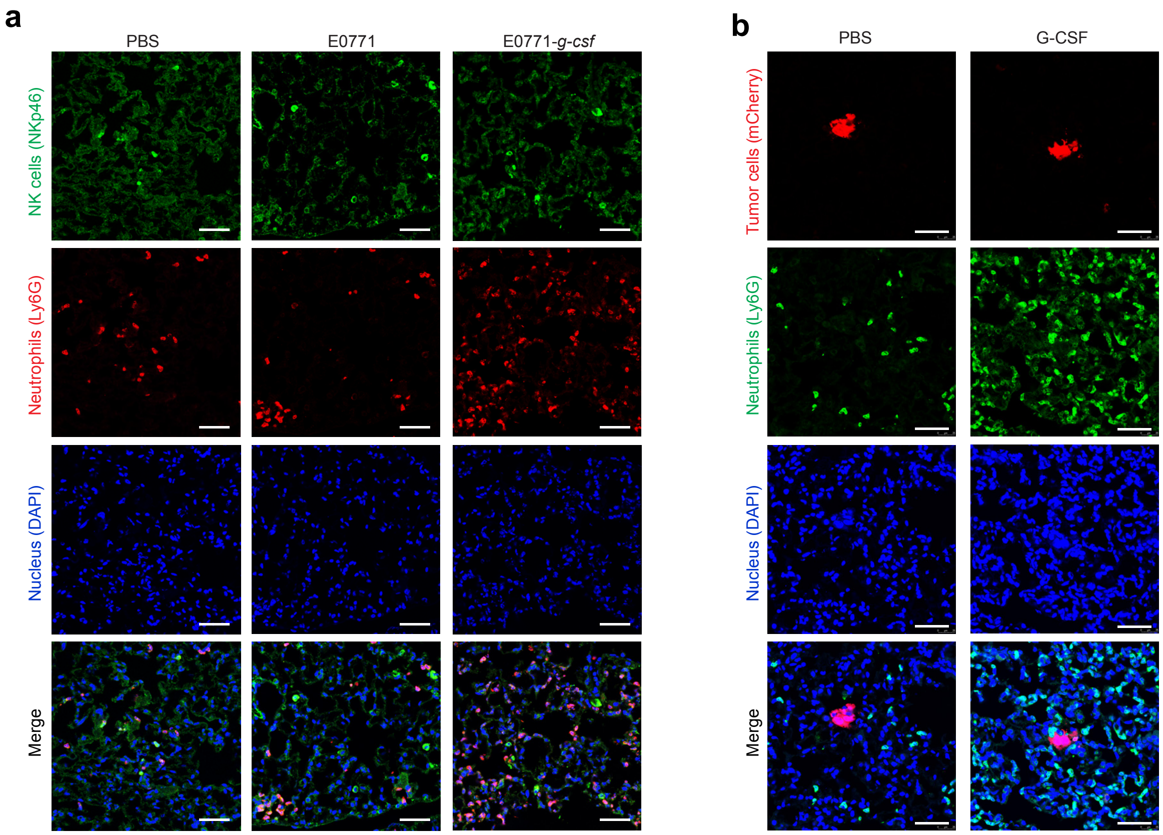

Supplementary Figure 7. Immunostaining of neutrophils, NK cells and tumor cells in lung sections from tumor-free and tumor-bearing mice

a, E0771 cells or E0771-*g-csf* cells were orthotopically implanted in NOD-*scid* mice, with PBS injection as a control. At the pre-metastatic stage (day 10), mouse lungs were freshly isolated and sectioned for immunostaining of neutrophils (Ly6G) and NK cells (NKp46). Representative pictures from 4 mice per group are shown. Two independent experiments were performed and similar results were obtained. Scale bars, 50 μm.

b, NSG mice received i.p. injection of PBS or recombinant mouse G-CSF (2.5 μg per mouse) for 7 consecutive days. On day 5, the mice were i.v. implanted with 4T1-mCherry cells. 2 days later, the mouse lungs were freshly isolated and sectioned for immunostaining of tumor cells (mCherry) and neutrophils (Ly6G). Representative pictures from 3 mice per group are shown. Two independent experiments were performed and similar results were obtained. Scale bars, 50 μm.

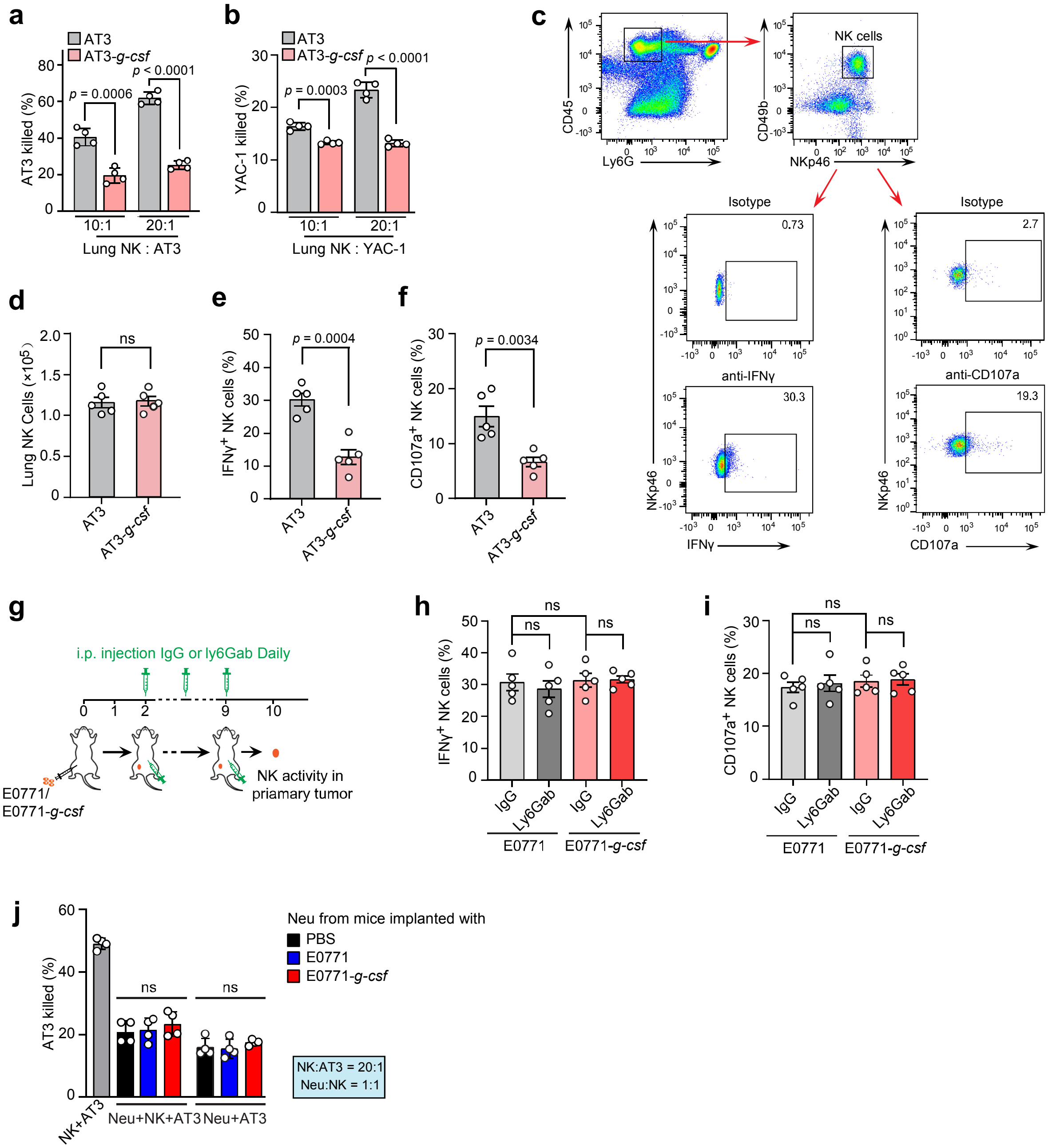

Supplementary Figure 8. Neutrophils suppress effector NK cells in the pre-metastatic lungs, but not in the primary tumors

a-b, d-f, AT3 cells and AT3-*g-csf* cells were orthotopically implanted in NOD-*scid* mice to induce non-inflammatory and inflammatory tumor-bearing host conditions, respectively. The mice were sacrificed at the pre-metastatic stage (day 10), and the lung-infiltrating NK cells were isolated for *ex vivo* cytotoxicity assay against AT3 (**a**) or YAC-1 cells (**b**). The total numbers of lung-

infiltrating NK cells were counted (**d**), and the percentages of IFNγ^+ (**e**) and CD107a$^+$ effector NK cells (**f**) were determined by flow cytometry.

c, Gating strategy to determine the percentages of IFNγ^+ and CD107a$^+$ cells within the NK cell population in mice, which corresponds to Figure 4b-d, Figure 4f-h, Figure 5b, Figure 6c-e, Supplementary Figure 8d-f, h-i, and Supplementary Figure 10b.

g-**i**, As depicted in (**g**), E0771 or E0771-*g-csf* cells were orthotopically implanted in NOD-*scid* mice, and the mice received control IgG or anti-Ly6G (12.5 μg per mouse) daily 2 days later after tumor cell implantation. At the pre-metastatic stage (day 10), the primary tumors were isolated and the percentages of IFNγ^+ (**h**) or CD107a$^+$ effector NK cells (**i**) were determined by flow cytometry.

j, Neutrophil-mediated tumoricidal effect, and their suppression of the tumoricidal activity of NK cells, were determined by *ex vivo* cytotoxicity assay against AT3 cells. Lung-infiltrating neutrophils were isolated from naive, E0771 and E0771-*g-csf* orthotopic tumor-bearing NOD-*scid* mice at their pre-metastatic stage (day 10). NK cells were derived from spleens of naïve NOD-*scid* mice.

Data are represented as mean ± SD of 4 biologically independent cell cultures (**a**, **b**, and **j**) or mean ± SEM (**d**-**f**, **h** and **i**, n = 5 mice per group), *P* values were determined by unpaired two-tailed *t*-test (**a**, **b** and **d**-**f**) or one-way ANOVA with Tukey's multiple comparisons test (**h**-**j**). ns, not significant. Source data are provided as a Source Data file.

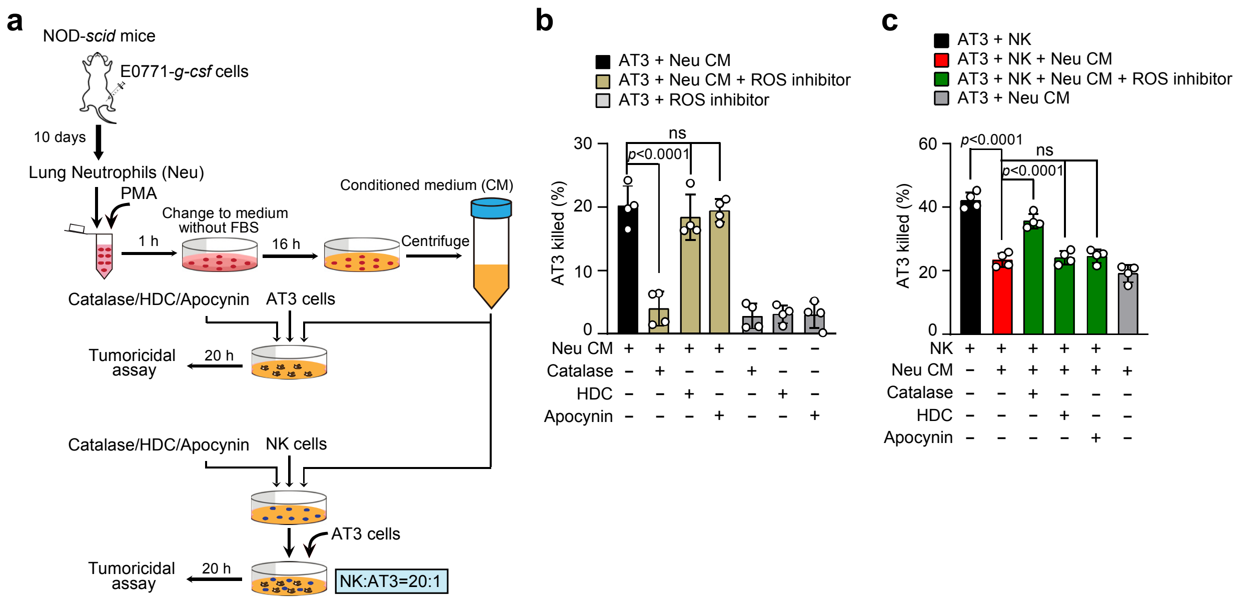

Supplementary Figure 9. Neutrophil-derived conditioned medium was effective in killing tumor cells and suppressing NK cells

As depicted in (**a**), neutrophils were freshly isolated from the lungs of E0771-*g-csf* orthotopic tumor-bearing NOD-*scid* mice, primed with PMA and cultured for another 16 hours for preparation of conditioned medium (CM). The CM was added into AT3 cell cultures (**b**) or NK cell cultures (**c**) in the absence and presence of ROS inhibitors catalase, HDC and apocynin. The *ex vivo* cytotoxicity assay against AT3 cells was then performed. NK cells were derived from spleens of naïve NOD-*scid* mice.

Data are represented as mean ± SD of 4 biologically independent cell cultures. *P* values were determined by one-way ANOVA with Tukey's multiple comparisons test. ns, not significant. Source data are provided as a Source Data file.

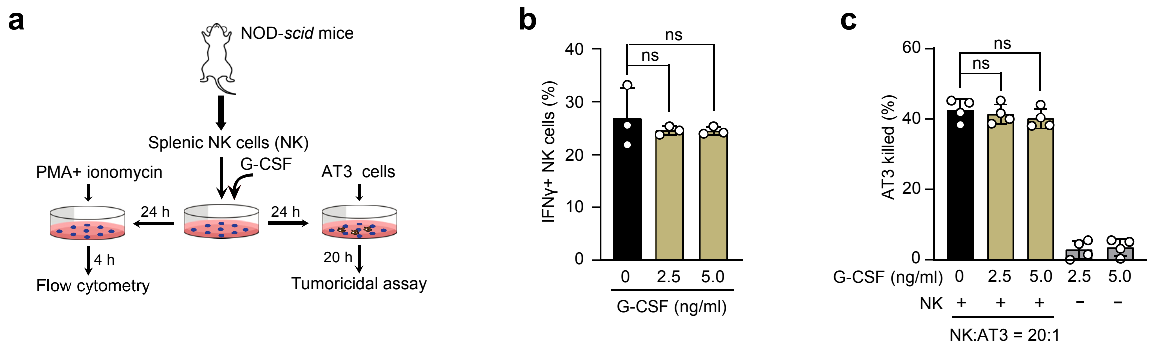

Supplementary Figure 10. The direct effect of G-CSF on NK cell functions

As depicted in (**a**), NK cells, derived from spleens of naïve NOD-*scid* mice, were first treated with recombinant mouse G-CSF at the indicated concentrations for 24 hours, and then subjected to determination of the percentages of IFNγ^+ NK cells by flow cytometry (**b**), or *ex vivo* cytotoxicity assay against AT3 cells (**c**).

Data are represented as mean ± SD of 3 (**b**) or 4 biologically independent cell cultures (**c**). Statistical significance was determined by one-way ANOVA with Tukey's multiple comparisons test, ns, not significant. Two independent experiments were performed and similar results were obtained. Source data are provided as a Source Data file.

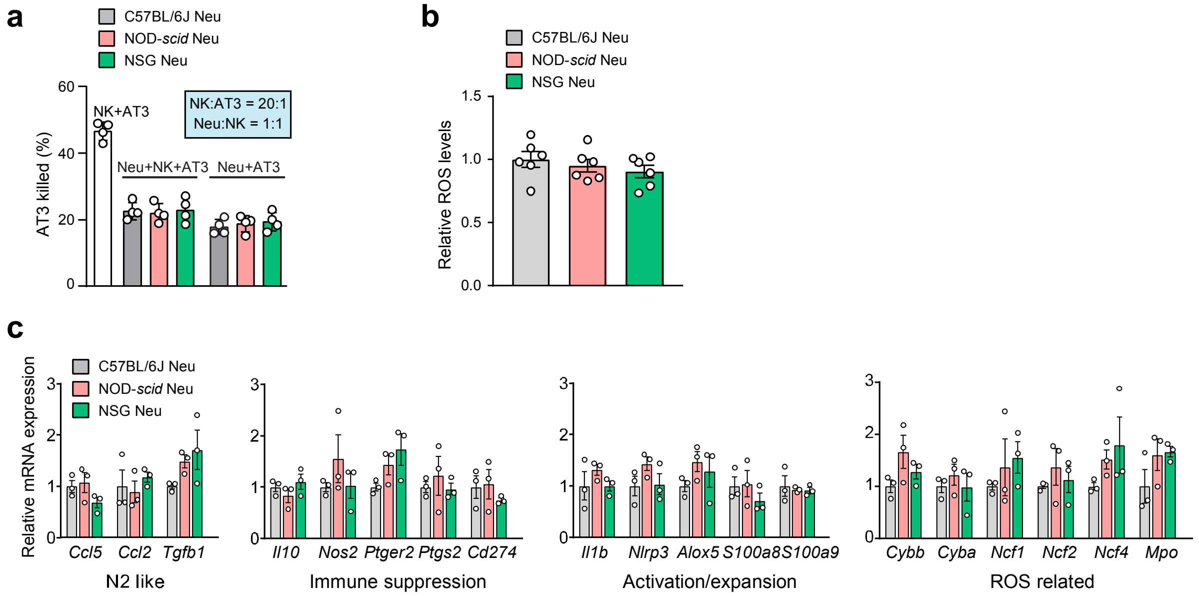

Supplementary Figure 11. Neutrophils isolated from NK-cell competent and NK cell-deficient mice exhibit similar characteristics

E0771-*g-csf* cells were orthotopically implanted in C57BL/6J, NOD-*scid* and NSG mice. At the pre-metastatic stage (day 10), the mouse lung neutrophils were isolated for determination of their suppressive effects on NK cells by *ex vivo* cytotoxicity assay against AT3 cells (**a**), and expression of the indicated groups of genes by q-PCR, with *Rps18* as the housekeeping gene (**c**). The sequences of primer were shown in Supplementary Table 1. The relative ROS levels in the lungs were detected by bioluminescence (**b**).

Data are represented as mean ± SD of 4 biologically independent cell cultures (**a**) or mean ± SEM (**b,** n = 6; **c**, n = 3 mice per group). Two independent experiments were performed and similar results were obtained. Source data are provided as a Source Data file.

Supplementary Table 1. Sequences of primer sets used in qRT-PCR

Gene	Gene bank accession NO.	Sequence (5'-3')
Rps18	NM_011296.3	GGAGAACTCACGGAGGATGA
		CCAGTGGTCTTGGTGTGCTG
Ccl5	NM_013653.3	TTTCTACACCAGCAGCAAGTGC
		TCCTTCGAGTGACAAACACGAC
Ccl2	NM_011333.3	TCTCTCTTCCTCCACCACCATG
		GCGTTAACTGCATCTGGCTGA
Tgfb1	NM_011577.2	TCACTGGAGTTGTACGGCAGTG
		TCGAAAGCCCTGTATTCCGTC
Il10	NM_010548.2	CCAAGCCTTATCGGAAATGA
		TTTTCACAGGGGAGAAATCG
Nos2	NM_010927.4	TGCCCCTTCAATGGTTGGTA
		ACTGGAGGGACCAGCCAAAT
Ptger2	NM_008964.4	CCTGCTGCTTATCGTGGCTG
		GCCAGGAGAATGAGGTGGTC
Ptgs2	NM_011198.4	TGCCTGGTCTGATGATGTATG
		GGGGTGCCAGTGATAGAGTG
Cd274	NM_021893.3	GCTCCAAAGGACTTGTACGTG
		TGATCTGAAGGGCAGCATTTC
Il1b	NM_008361.4	ACCTTCCAGGATGAGGACATGA
		CTAATGGGAACGTCACACACCA
Nlrp3	NM_001359638.1	AGAGCCTACAGTTGGGTGAAATG
		CCACGCCTACCAGGAAATCTC
Alox5	NM_009662.2	CTCTTCCAAGCTCGAAGTGC
		TGATGCTACCGAGTGACGAG
S100a8	NM_013650.2	GGAAATCACCATGCCCTCTACAA
		ATGCCACACCCACTTTTATCACC
S100a9	NM_001281852.1	GGAGCGCAGCATAACCACCATC
		GCCATCAGCATCATACACTCCTCA
Cybb	NM_007807.5	TTCTTCATCGGCCTTGCCAT
		GCCAAAACCGAACCAACCTC
Cyba	NM_007806.3	CCTCCACTTCCTGTTGTCGG
		TCACTCGGCTTCTTTCGGAC
Ncf1	NM_001286037.1	TGGAGGGCAGAGACAATCCA
		AGGGATAGGAGCCGTCTAGG
Ncf2	NM_010877.5	CTATCTGGGCAAGGCTACGG
		GCCCAGTTATCACTGCCCTT
Ncf4	NM_008677.2	GCGCAAAATCAAGGGTGTGT
		ATCGTAGCCAGTTGGTGGTG
Mpo	NM_010824.2	AACATGCAGCGCAGCCGG
		AGCCCACAAAAGCGTCTC

Supplementary References

1. Ishikawa F*, et al.* Development of functional human blood and immune systems in NOD/SCID/IL2 receptor {gamma} chain(null) mice. *Blood* **106**, 1565-1573 (2005).

2. Shultz LD*, et al.* Human lymphoid and myeloid cell development in NOD/LtSz-scid IL2R gamma null mice engrafted with mobilized human hemopoietic stem cells. *Journal of immunology* **174**, 6477-6489 (2005).
