# Supplementary material for: Dual roles of neutrophils in metastatic colonization are governed by the host NK cell status
Source: Nat Commun. 2020 Sep 1;11:4387. doi: 10.1038/s41467-020-18125-0 (PMC7463263; doi:10.1038/s41467-020-18125-0)
Supplement: Supplementary file 3 — Reporting Summary [file 41467_2020_18125_MOESM3_ESM.pdf]

nature research

Corresponding author(s): Guangwen Ren

Last updated by author(s): Jul 23, 2020

Reporting Summary

Nature Research wishes to improve the reproducibility of the work that we publish. This form provides structure for consistency and transparency in reporting. For further information on Nature Research policies, see Authors & Referees and the Editorial Policy Checklist .

Statistics

For all statistical analyses, confirm that the following items are present in the figure legend, table legend, main text, or Methods section.

n/a	Confirmed	
☐	☒	The exact sample size (*n*) for each experimental group/condition, given as a discrete number and unit of measurement
☐	☒	A statement on whether measurements were taken from distinct samples or whether the same sample was measured repeatedly
☐	☒	The statistical test(s) used AND whether they are one- or two-sided<br>*Only common tests should be described solely by name; describe more complex techniques in the Methods section.*
☒	☐	A description of all covariates tested
☒	☐	A description of any assumptions or corrections, such as tests of normality and adjustment for multiple comparisons
☐	☒	A full description of the statistical parameters including central tendency (e.g. means) or other basic estimates (e.g. regression coefficient) AND variation (e.g. standard deviation) or associated estimates of uncertainty (e.g. confidence intervals)
☐	☒	For null hypothesis testing, the test statistic (e.g. *F*, *t*, *r*) with confidence intervals, effect sizes, degrees of freedom and *P* value noted<br>*Give P values as exact values whenever suitable.*
☒	☐	For Bayesian analysis, information on the choice of priors and Markov chain Monte Carlo settings
☒	☐	For hierarchical and complex designs, identification of the appropriate level for tests and full reporting of outcomes
☒	☐	Estimates of effect sizes (e.g. Cohen's *d*, Pearson's *r*), indicating how they were calculated

Our web collection on statistics for biologists contains articles on many of the points above.

Software and code

Policy information about availability of computer code

Data collection	BD FACSDiVa software (version 8) was used for flow cytometry acquisition. Xenogen IVIS image system was used for bioluminescence image collection. Leica Application Suite X (LAS X) software platform was used for image capture from Leica microscopes.
Data analysis	Graphpad Prism 7.04 was used for data analysis. FlowJo 10.1 was used for preprocessing of the flow cytometry data and analysis.

For manuscripts utilizing custom algorithms or software that are central to the research but not yet described in published literature, software must be made available to editors/reviewers. We strongly encourage code deposition in a community repository (e.g. GitHub). See the Nature Research guidelines for submitting code & software for further information.

Data

Policy information about availability of data

All manuscripts must include a data availability statement. This statement should provide the following information, where applicable:

- Accession codes, unique identifiers, or web links for publicly available datasets
- A list of figures that have associated raw data
- A description of any restrictions on data availability

The source data underlying Figs. 1b-d, 1f, 1g, 2b, 2d-g, 2i, 2j, 3c, 3e-i, 4b-d, 4f-h, 4j, 4k, 5b-f, 6b-e, 6g, 6i and Supplementary Figs. 1b, 1c, 1e, 2a, 2b, 4a-i, 5b-d, 6b, 8a, 8b, 8d-f, 8h-j, 9b, 9c, 10b, 10c, 11a-c are provided as a Source Data file. All the other data supporting the findings of this study are available within the article and its supplementary information files and from the corresponding author upon reasonable request.

Field-specific reporting

Please select the one below that is the best fit for your research. If you are not sure, read the appropriate sections before making your selection.

Life sciences study design

All studies must disclose on these points even when the disclosure is negative.

Sample size	No statistical method was used to predetermine sample size. For ex vivo/in vitro experiments, 3-4 biological replicates were used and this sample size is sufficient for a statistical analysis. For in vivo experiments, a sample size of n = 4-10 mice was used per experimental group. This sample size was determined based on the level of expected heterogeneity of the samples, the significance threshold (chosen at 0.05), the expected or observed difference, as well as the previous publications (PMIDs: 23168163; 25203322; 27072748; 26649828; and 25822788) and our pilot studies for each experiment. Based on our previous studies (PMIDs: 23168163 and 25203322), the chosen sample size in each experiment is sufficient to generate statistically significant results.
Data exclusions	No data were excluded from the manuscript.
Replication	All in vivo, ex vivo and in vitro data are representative of two to three independent experiments. The information is included in the “Statistics and Reproducibility” in the methods section.
Randomization	All mice were randomized before tumor inoculation.
Blinding	Except the immunostaining evaluation (Supplementary Figure 2C & 7), all other experiments were unblinded because these analyses were performed using quantifiable parameters and no bias was involved. For example, photon flux of luciferase signal values were used for monitoring the lung metastasis; the luminescence values detected by luminometer were used for in vitro cytotoxic assay; and the values of percentage or number of NK+ cells were derived from flowJo analysis using the same gating strategy.

Reporting for specific materials, systems and methods

We require information from authors about some types of materials, experimental systems and methods used in many studies. Here, indicate whether each material, system or method listed is relevant to your study. If you are not sure if a list item applies to your research, read the appropriate section before selecting a response.

Materials & experimental systems

n/a	Involved in the study	
[ ]	[x]	Antibodies
[ ]	[x]	Eukaryotic cell lines
[x]	[ ]	Palaeontology
[ ]	[x]	Animals and other organisms
[x]	[ ]	Human research participants
[x]	[ ]	Clinical data

Methods

n/a	Involved in the study	
[x]	[ ]	ChIP-seq
[ ]	[x]	Flow cytometry
[x]	[ ]	MRI-based neuroimaging

Antibodies

Antibodies used

All of the antibodies used for flow cytometry were purchased from Biolegend, and the dilution for antibodies used in flow cytometric analysis is 1:200.
(molecule, fluorophore, Clone, Catalog #, company)
CD45, Alexa Fluor 700, 30-F11, 103128, BioLegend
B220, BUV496, RA3-6B2, 612950, BD Biosciences
CD4, PE-Cy5, GK1.5, 100410, BioLegend
CD8, BV650, 53-6.7, 100742, BioLegend
CD19, AF647, 6D5, 115522, BioLegend
CD90.2, AF647, 30-H12, 105318, BioLegend
Siglec F, AF647, E50-2440, 562680, BD Biosciences
CD11c, Percp-Cy5.5, N418, 117328, BioLegend
CD24: PE-Cy7, M1/69, 101822, BioLegend
CD3e, PerCP/Cy5.5, 145-2C11, 100328, BioLegend
CD49b, PE, DX5, 108908, BioLegend
NKp46, APC, 29A1.4, 137608, BioLegend
CD107a, BV510, 1D4B, 121629, BioLegend
IFN-γ, FITC, XMG1.2, 505805, BioLegend
CD11b, BV650, M1/70, 101239, BioLegend
Ly6c, BV570, HK1.4, 128030, BioLegend
Ly6G, Pacific Blue, 1A8, 127611, BioLegend
MHC class II, APC/Cy7, M5/114.15.2, 107627, BioLegend

The antibodies used for animal injection were purchased from BioXCell: Rat IgG (InVivoPlus rat IgG2a isotype control, anti-

trinitrophenol, Clone 2A3, Catalog #BP0089), anti-Ly6G (InVivoMAb anti-mouse Ly6G, Clone 1A8, Catalog #BE0075-1), Mouse IgG (InVivoMAb mouse IgG2a isotype control, unknown specificity, Clone C1.18.4, Catalog #BE0085), anti-NK1.1 (InVivoMAb anti-mouse NK1.1, Clone PK136, Catalog # BE0036).
The antibody used for NK cell depletion in NOD mice were purchased from Biolegend, anti-Asialo-GM1 (Ultra-LEAF™ Purified anti-Asialo-GM1 Antibody, Clone Poly21460, Catalog # 146002).
The antibodies used for immunostaining were purchased from Thermo Fisher: Ly-6G/ly6c Monoclonal Antibody (clone: RB6-8C5) (Catalog #14-5931-81); NKp46 Polyclonal Antibody(Catalog # PA5-79720); Goat anti-Rat IgG (H+L) Cross-Adsorbed Secondary Antibody, Alexa Fluor 488 (Catalog # A-11006); Goat anti-Rat IgG (H+L) Cross-Adsorbed Secondary Antibody, Alexa Fluor 555 (Catalog # A-21434); Goat anti-Rabbit IgG (H+L) Highly Cross-Adsorbed Secondary Antibody, Alexa Fluor Plus 488 (Catalog # A32731).

Validation

Validations of antibodies, including assay and species were provided in data sheets from Research Resource Identifier (RRID) or the manufacturer's website. The detailed information are listed as follows:

CD45 (Alexa Fluor 700, 30-F11, 103128, BioLegend)
RRID:AB_493715, cited from RRID website at: https://antibodyregistry.org/search.php?q=AB_493715%3c/result%3e

B220 (BUV496, RA3-6B2, 612950, BD Biosciences)
RRID:AB_2722578, cited from RRID website at: https://antibodyregistry.org/search.php?q=AB_2722578

CD4 (PE-Cy5, GK1.5, 100410, BioLegend)
RRID:AB_312697, cited from RRID website at: https://antibodyregistry.org/search.php?q=AB_312697

CD8 (BV650, 53-6.7, 100742, BioLegend)
RRID:AB_11124344, cited from RRID website at: https://antibodyregistry.org/search.php?q=AB_11124344%3c/result%3e

CD19 (AF647, 6D5, 115522, BioLegend)
RRID:AB_389329, cited from RRID website at: https://antibodyregistry.org/search.php?q=AB_389329%3c/result%3e

CD90.2(AF647, 30-H12, 105318, BioLegend)
RRID:AB_492888, cited from RRID website at: https://antibodyregistry.org/search.php?q=AB_492888%3c/result%3e

Siglec F (AF647, E50-2440, 562680, BD Biosciences)
RRID:AB_2687570, cited from RRID website at: https://antibodyregistry.org/search.php?q=AB_2687570

CD11c (Percp-Cy5.5, N418, 117328, BioLegend)
RRID:AB_2129641, cited from RRID website at: https://antibodyregistry.org/search.php?q=AB_2129641

CD24 (PE-Cy7, M1/69, 101822, BioLegend)
RRID:AB_2832499, cited from RRID website at: https://antibodyregistry.org/search.php?q=AB_2832499

CD3e (PerCP/Cy5.5, 145-2C11, 100328, BioLegend)
RRID:AB_893318, cited from RRID website at: https://antibodyregistry.org/search.php?q=AB_893318

CD49b (PE, DX5, 108908, BioLegend)
RRID:AB_313415, cited from RRID website at: https://antibodyregistry.org/search.php?q=AB_313415

NKp46 (APC, 29A1.4, 137608, BioLegend)
RRID:AB_10612758, cited from RRID website at: https://antibodyregistry.org/search.php?q=AB_10612758

CD107a (BV510, 1D4B, 121629, BioLegend)
RRID:AB_2783064, cited from RRID website at: https://antibodyregistry.org/search.php?q=AB_2783064%3c/result%3e

IFN-γ (FITC, XMG1.2, 505805, BioLegend)
RRID:AB_315400, cited from RRID website at: https://antibodyregistry.org/search.php?q=AB_315400

CD11b (BV650, M1/70, 101239, BioLegend)
RRID:AB_11125575, cited from RRID website at: https://antibodyregistry.org/search.php?q=AB_11125575%3c/result%3e

Ly6c (BV570, HK1.4, 128030, BioLegend)
RRID:AB_2562617, cited from RRID website at: https://antibodyregistry.org/search.php?q=AB_2562617%3c/result%3e

Ly6G (Pacific Blue, 1A8, 127611, BioLegend)
RRID:AB_1877212, cited from RRID website at: https://antibodyregistry.org/search.php?q=AB_1877212%3c/result%3e

MHC class II (APC/Cy7, M5/114.15.2, 107627, BioLegend)
RRID:AB_1659252, cited from RRID website at: https://antibodyregistry.org/search.php?q=AB_1659252

Rat IgG (InVivoPlus rat IgG2a isotype control, anti-trinitrophenol, Clone 2A3, Catalog #BP0089, BioXCell)
RRID:AB_1107769, cited from RRID website at: https://antibodyregistry.org/search.php?q=AB_1107769

anti-Ly6G (InVivoMAb anti-mouse Ly6G, Clone 1A8, Catalog #BE0075-1, BioXCell)
RRID:AB_1107721, cited from RRID website at: https://antibodyregistry.org/search.php?q=AB_1107721

Mouse IgG (InVivoMAb mouse IgG2a isotype control, unknown specificity, Clone C1.18.4, Catalog #BE0085, BioXCell)
RRID:AB_1107771, cited from RRID website at: https://antibodyregistry.org/search.php?q=AB_1107771

anti-NK1.1 (InVivoMAb anti-mouse NK1.1, Clone PK136, Catalog # BE0036, BioXCell)
RRID:AB_1107737, cited from RRID website at: https://antibodyregistry.org/search.php?q=AB_1107737

anti-Asialo-GM1 (Ultra-LEAF™ Purified anti-Asialo-GM1 Antibody, Clone Poly21460, Catalog # 146002,Biolegend)
RRID:AB_2562206, cited from RRID website at: https://antibodyregistry.org/search.php?q=AB_2562206%3c/result%3e

NKp46 Polyclonal Antibody (Catalog # PA5-79720, Thermo Fisher)
RRID: AB_2746835, cited from RRID website at: https://antibodyregistry.org/search.php?q=AB_2746835

Ly-6G/ly6c Monoclonal Antibody (clone: RB6-8C5, Catalog #14-5931-81, Thermo Fisher)
RRID: AB_467729, cited from RRID website at: https://antibodyregistry.org/search.php?q=AB_467729

Goat anti-Rat IgG (H+L) Cross-Adsorbed Secondary Antibody, Alexa Fluor 488 (Catalog # A-11006, Thermo Fisher)
RRID: AB_141373, cited from RRID website at: https://antibodyregistry.org/search.php?q=AB_141373

Goat anti-Rat IgG (H+L) Cross-Adsorbed Secondary Antibody, Alexa Fluor 555 (Catalog # A-21434, Thermo Fisher)
RRID: AB_2535855, cited from RRID website at: https://antibodyregistry.org/search.php?q=AB_2535855

Goat anti-Rabbit IgG (H+L) Highly Cross-Adsorbed Secondary Antibody, Alexa Fluor Plus 488 (Catalog # A32731, Thermo Fisher)
RRID: AB_2633280, cited from RRID website at: https://antibodyregistry.org/search.php?q=AB_2633280

Eukaryotic cell lines

Policy information about cell lines

Cell line source(s)	E0771 cell line were purchased from CH3 Biosystems; AT3 cell line was originally generated from Dr. Scott I. Abrams's group at Roswell Park Comprehensive Cancer Center (J Immunol 179: 2851–2859. 2007); 4T1 and YAC-1 cell lines were purchased from American Type Culture Collection (ATCC).
Authentication	Cells were newly acquired from above sources. No further validation was preformed.
Mycoplasma contamination	All cell lines were all tested negative for mycoplasma contamination.
Commonly misidentified lines (See ICLAC register)	No commonly misidentified cell lines were used.

Animals and other organisms

Policy information about studies involving animals; ARRIVE guidelines recommended for reporting animal research

Laboratory animals	C57BL/6J (JAX stock #000664), BALB/c (JAX stock #000651), NOD.Cg-Prkdcscid/J (NOD-scid, JAX stock #001303), B6.Cg-Prkdcscid/SzJ (B6-scid, JAX stock #001913) and NOD.Cg-PrkdcscidIl2rgtm1Wjl/SzJ (NSG, JAX stock #005557) mice were obtained from the Jackson Laboratory. The mice were fed on a chow diet ad libitum and housed in a specific pathogen-free facility in plastic cages at 22 °C and 40-50% humidity, with a daylight cycle from 6 a.m. to 6 p.m. All mice used in the manuscript were females between 8-10 weeks old at the moment of tumor inoculation.
Wild animals	Our study did not involve wild animals.
Field-collected samples	Our study did not involve samples collected from the field.
Ethics oversight	Animal protocols were reviewed and approved by the Institutional Animal Care and Use Committee of the Jackson Laboratory.

Note that full information on the approval of the study protocol must also be provided in the manuscript.

Flow Cytometry

Plots

Confirm that:

- [x] The axis labels state the marker and fluorochrome used (e.g. CD4-FITC).
- [x] The axis scales are clearly visible. Include numbers along axes only for bottom left plot of group (a 'group' is an analysis of identical markers).
- [x] All plots are contour plots with outliers or pseudocolor plots.
- [x] A numerical value for number of cells or percentage (with statistics) is provided.

Methodology

Sample preparation	For NK cell surface marker analysis, cells were suspended in staining buffer (PBS, 2% FBS) at a concentration of less than 2 x 10e7 cells/ml and 100 ul of suspension was incubated with fluorochrome -conjugated CD45, CD3e, CD49b, NKp46, Ly6G, and CD107a antibodies (Biolegend, San Diego, CA) for 30 min on ice. Cells were washed twice with staining buffer. Fluorescence intensity was measured by flow cytometry (FACSymphony A5, BD Immunocytometry, San Jose, CA). For detection of intracellular IFNg, cells were primed with PMA (100 ng/ml) and ionomycin (1 ug/ml) for 4 hrs. Then the cells were fixed after surface staining, permeabilized with Cytofix/Cytoperm (BD Biosciences, San Jose, CA) and stained with an anti-IFNg antibody (Biolegend, San Diego, CA). Data were analyzed using FlowJo software.
Instrument	FACSymphony A5, BD Immunocytometry
Software	Data were recorded with FACSDiva software and analyzed by FlowJo 10.1
Cell population abundance	Neutrophils were purified using anti-Ly6G magnetic beads (Miltenyi Biotech, Auburn, CA) according to the manufacturer’s instructions. The purity for neutrophils is over 95% as tested by flow cytometry. NK cells were purified from splenic cells using the NK cell isolation kit (Miltenyi Biotech, Auburn, CA) or from lung dissociated cells using the CD49b (DX5) MicroBeads (Miltenyi Biotech, Auburn, CA). The purity for NK cells is over 90% as tested by flow cytometry.
Gating strategy	The cells were gated using FSC-A and SSC-A followed by single cell discrimination using FSC-A and FSC-H. DAPI negative was used to gate for viable cells. The isotype control antibody was used to set up the gates.

- [x] Tick this box to confirm that a figure exemplifying the gating strategy is provided in the Supplementary Information.
